# Can the Participation in Quality Certification of Agricultural Products Drive the Green Production Transition?

**DOI:** 10.3390/ijerph191710910

**Published:** 2022-09-01

**Authors:** Xiaojing Li, Xianli Xia, Jiazhen Ren

**Affiliations:** 1 School of Economics and Management, Yantai University, Yantai 264005, China; 2 College of Economics and Management, Northwest Agriculture and Forestry University, Yangling, Xianyang 712100, China

**Keywords:** quality certification, kiwifruit growers, green production transition, finite mixture model

## Abstract

The green production transition in agriculture is all about the quality of agricultural products at the source of production. Whether the product quality certification can accelerate the green production transition in agriculture is an issue of concern. We have measured the degree of green production transition of kiwifruit growers using a finite mixture model in this paper, and use research data from the main kiwifruit production areas in Shaanxi and Sichuan provinces to verify the impact of conducting product quality certification on the green production transition of kiwifruit growers. Besides, we use a multi-valued treatment effects model to verify the differences in the degree of green production transition among kiwifruit growers in the face of different certification types. Our findings are mainly as follows: the degree of green production transition among kiwifruit growers is not high, with an average of only 36.3%. Product quality certification can significantly promote the green production transition of kiwifruit growers, and the promotion effect of different certification methods in green production transition of kiwifruit growers significantly varies. The promotion effect of organic certification is greater than that of green certification and pollution-free certification. Further, the mechanism test analysis reveals that product quality certification can influence the green production transition of kiwifruit growers through three mechanisms: quality monitoring, market premium, and market access threshold. Based on this, this paper proposes policy recommendations to advance quality certification and green production transition among kiwifruit growers to increase the certification, enhance the willingness to green transition, and boost the differentiated certification system.

## 1. Introduction

In 2020, China used 52,507,000 tons of chemical fertilizers (discounted stock), about twice as much as in 1990, and 1,133,000 tons of pesticides in the same period, about 1.8 times as much as in 1900. The excessive application of chemical fertilizers and pesticides has increased the production per unit area in the short-term, but in the long-term it has brought a series of problems such as the decreasing utilization of water and soil resources, serious pollution of agricultural surface sources, and increasing the hidden dangers of agricultural product quality and safety. These problems have further triggered conflicts between population, resources, environment, and economic development, and become the main obstacle that restricts China’s comprehensive, coordinated, and sustainable socio-economic development and continuously meeting the people’s growing needs for a better life. Therefore, it is imperative to accelerate the transformation of the agricultural development mode and implement the transformation of agricultural green production.

The transformation of agricultural green production depends on the quality of the source of agricultural production, and whether implementing product quality certification can accelerate the transformation of agricultural green production is a matter of concern. Since 2012, the Chinese government has made significant decisions and arrangements to gradually advance the agricultural product quality certification. However in practice, there are a series of significant problems plaguing the development of green agriculture, such as failure by the farmers to observe the standard of “discounted” production, excessive use of chemical fertilizers and pesticides, and illegal sales of substandard products [1,2,3]. These show that the government’s efforts in upgrading agricultural quality certification for the time being are more complex and challenging for Chinese agriculture as in the transition of greening [4,5].

Some scholars have explored the impact of agricultural quality certification on agricultural producers. Geng et al. [6] found that certified agricultural products have a significant price advantage. The certified growers will actively adopt green production techniques and minimize the pesticides and fertilizers that are applied to their agricultural products to comply with product quality certification standards, thus improving the quality of their agricultural products and generating higher sales revenue at higher transaction prices [7,8]. Using the research data from vegetable growing areas, Li and Lu [9] found that farmers’ technical efficiency largely depends on farmers’ certification of organic agricultural products and green food certification. In addition, Tran and Goto [5] investigated that farmers’ quality certification of agricultural products can significantly improve the selling price and increase their economic returns. Even though the large number of certification funds that is required for certification causes farmers to shoulder higher production costs, the advantageous quality certification can help them access more modern retail channels and thus increase the total sales of agricultural products [10]. The existing studies have laid a certain theoretical foundation for this study. However, there are still the following issues that are worthy of further discussion. Firstly, few studies have analyzed in depth the impact of participation in product quality certification on the transition of green agricultural production of farmers, and even fewer have paid attention to its intrinsic impact mechanism. Secondly, most of them have attached importance to the green production transition of producers in industrial enterprises, although a series of results have been achieved in academic research in this respect. Moreover, fewer research results on the green production transition of agricultural producers were seen. Thirdly, the existing literature mainly explored the mechanism of farmers’ participation in product quality certification from the perspective of single product quality certification, rather than its effect on producers’ green production transition from multiple product quality certifications.

The remainder of this article is organized as follows. In the Section 2, we have built a framework under which the impact of participating in product quality certification on farmers’ green agricultural production transition is further discussed. The Section 3 introduces our estimation approaches, identification strategies, and the data that were used in this study. The Section 4 shows the main estimation results and discussion based upon our empirical estimation. The Section 5 concludes our findings and highlights the policy implications.

## 2. Theoretical Framework and Research Hypothesis

### 2.1. Product Quality Certification and Quality Monitoring

Subject to the typical trust attributes of agricultural products, it is difficult for consumers to precisely identify whether the production process of agricultural products is safe and hygienic before and after purchase [11]. As an essential means to disclose the quality information of agricultural products, the product quality certification system is a powerful initiative to lower the quality and safety risks of agricultural products [12,13]. It transmits safety information to consumers by affixing certification marks on agricultural products. With the help of the marks, the trust attributes of agricultural products can be transformed into a search attributes [14]. Producers with product quality certification are subject to pre-sale testing, traceability codes, and quality control of their agricultural products following the certification standards. These efforts are made to provide relevant certification information to facilitate consumers’ screening and purchase [15,16]. Since consumers always keep an eye on the quality of agricultural products, to ensure the quality of and improve the credibility of agricultural products [17,18], farmers will favor green production technology to improve production capacity and quality of products and then force their green production transition [19]. In addition, the agricultural quality certification registration system in China caps a strict validity period for producers to entitle the certification status [20]. Suppose the producers are found to use prohibited pesticides or fail to meet the standards in the sampling inspection of agricultural products during the annual inspection, the farmers who have been certified will be subject to the penalty of being disqualified for using the certification [21]. In contrast, those with quality certification will intensify their control over the quality of agricultural products to prevent their certification being terminated [22]. Producers of agricultural products take the initiative to comply with the certification standards for production [23], which will prompt them to gradually shift from the extensive production to the green one, thus guaranteeing a high-quality supply of agricultural products and ultimately realizing their green production transition.

**H1:**  *Product quality certification boosts the transition of green agricultural production by improving the level of quality monitoring.*

### 2.2. Product Quality Certification and Product Premium

The high-value functional attributes of the product that are brought about by quality certification can cater to consumers’ purchasing needs, and their ability to afford the “trust premium” for agricultural products will gradually increase. It is possible to sharpen farmers’ competitive edge and boost their bargaining power by participating in product quality certification [24], thereby significantly increasing the sales price of green agricultural products and driving them to transform their production methods to green production [25]. Green agricultural products effectively stimulate producers to shift from price competition to quality competition, significantly increasing the profitability of agricultural production [26]. At this time, the improvement in profitability will strengthen farmers’ willingness to reinvest both capital and technology in green agricultural products, forming a virtuous cycle of “input-profit-reinvestment”, which in turn will propel farmers’ transition of production methods to green production [27]. In addition, as consumer brand awareness rises, consumers have a higher preference for the green attributes of agricultural products and the attention to and enthusiasm for pollution-free, green, and organic food will increase with each passing day [28,29]. Agricultural producers will be strongly motivated by external market economic income to actively change the original backward and sloppy production methods and turn to those greener production methods of higher quality to achieve green production transition and pursue higher economic income [30]. Producers will in this way supply more high-quality green agricultural products and obtain value-added income from consumers’ green preferences [31].

**H2:**  *Product quality certification propels the transition of green agricultural production by increasing product premiums.*

### 2.3. Product Quality Certification and Market Access Barrier

**Figure 1:**
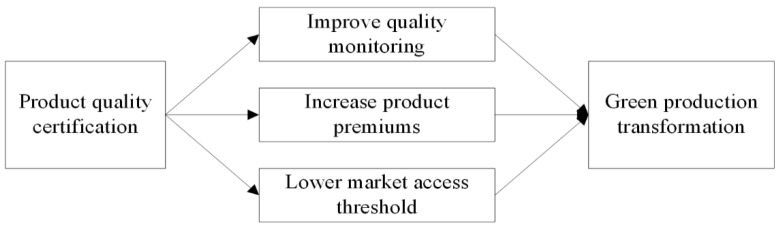
The pathway underlying the impact of product quality certification on the transition of green production.

Product quality certification provides a “credit card” or “green pass” for green agricultural products to enter modern markets, helping to reduce consumers’ perception of uncertainty about product quality and thus increase their willingness to buy [32,33]. The market share of certified products can be increased by lowering the higher market barriers arising from information asymmetry, while improving farmers’ economic returns in the marketing chain [34,35]. Benefiting from the increased returns, farmers’ willingness to increase investment in green technologies will be significantly enhanced and their yearn for green production transition will be strengthened as well [36]. In order to comply with production standards, farmers with certified product quality will take the initiative to increase production-specific investments and upgrade product technologies for the purpose of levelling the degree of product differentiation [37]. The higher technology level of agricultural products will uniquely advantage the products, which will help to lower the access barrier to entering the broader markets [38,39]. Traditional distribution channels—mainly wholesale markets—did not set higher quality standards for agricultural products. Modern channels, such as supermarkets and Internet-based distribution markets, in pursuit of competitive advantages in product quality, however encourage farmers to improve the quality of their agricultural products by setting more stringent industry quality standards [40]. This incentivizes farmers to actively transition to green production and makes for their quality products to access higher-end supermarkets and online shopping malls. A brief theoretical framework is presented in Figure 1. Therefore, we propose the following hypotheses for this study.

**H3:**  *Product quality certification pushes the transition of green agricultural production by lowering the threshold of market access.*

**H4:**  *The degree of green production transition of farmers participating in quality certification is higher than that of those who do not.*

## 3. Methods

The empirical analysis in this paper is based on the ideas below. First, the heterogeneity of kiwifruit growers’ production methods was judged according to a finite mixture model, and kiwifruit growers with different production methods were classified into different potential categories. Second, the probability of green production transition of kiwifruit growers was measured based on the categories. Finally, the effect of different certification types on the probability of green production transition of kiwifruit growers was analyzed using the multi-value treatment effect value to analyze the relationship between different certification types and green production transition.

### 3.1. Finite Mixture Model

Considering the existence of different potential categories of growers in terms of the choice of production methods (traditional production category and green production category), we referred to the ways by which some scholars have studied the potential categories of samples, the distribution function of the full sample was expressed as several sub-probability density functions [41], one of which is expressed as follows:

(1) $$f(Y|X,\theta )=\sum_{k=1}^{K}\pi_{k}f(Y|X,\theta_{k})=\pi_{1}f_{1}(X)+\pi_{2}f_{2}(X)+\cdots \pi_{k}f_{k}(X)$$

where, $f(Y|X,\theta_{k})$ denotes the conditional density distribution of Y falling under the potential category k due to unobservable heterogeneity factors; X is a vector that is composed of explanatory variables; $\theta_{k}$ is the parameter to be estimated; and $\pi_{k}$ is denoted as the mixing ratio, and $\sum \pi_{k}=1$. 

By calculating Equation (2), the posterior probability of each sample falling into the first category can be calculated, thus classifying different samples under different potential categories. Since green production transition is not a precise technique but a comprehensive state, the posterior probability of a sample falling into the category of green production practices expresses, to some extent, the degree of green production transition of growers.

(2) $$P(j|X,Y)=\frac{\pi_{j}f_{j}(Y|X,\theta_{j})}{\sum_{k}\pi_{k}f_{k}(Y|X,\theta_{k})}$$

### 3.2. Multiple Linear Regression Model

To examine the effect of product quality certification on the green production transition of kiwifruit growers, a multiple linear regression model was used for the baseline regression because the degree of green production transition is a continuous variable [42]. The following model was therefore constructed:

(3) $$y=\beta_{0}+\alpha_{1}x_{1}+\sum_{i=1}^{k}\beta_{i}Control_{i}+\mu_{i}$$

where, y is the explained variable, representing the degree of green production transition; $x_{1}$ is the explanatory variable, representing whether or not product quality certification is performed; $Control_{i}$ is the control variable; $\mu_{i}$ is the residual term; and $\alpha_{1},\beta_{i}$ is the parameter to be estimated.

### 3.3. Conditional Mixed Estimation Model (CMP)

Considering the selectivity bias due to observable and unobservable factors, the instrumental variables approach was used to address the endogeneity in the impact of product quality certification on the green production transition by kiwifruit growers. The endogenous variable product quality certification is a binary dummy variable, and the IV-Probit model and 2SLS model can only solve the case where the endogenous variable is a continuous variable. As a result, we mainly employed the CMP methods to cope with the endogeneity [43]. CMP estimation involved two steps. One was to find the instrumental variables that are the core endogenous explanatory variables in the empirical model and thus discerned their correlation [44]. The second was to bring the instrumental variables into the baseline model for regression and judge their homogeneity based on the significance of the endogeneity test parameter (atanhrho_12). If the endogeneity test parameter is significantly non-zero, it indicates that the instrumental variables that are selected are appropriate, and the estimation results of the CMP are better than those of the baseline model [45]. We applied this method to estimate the decision equation for product quality certification and for the degree of green production transition, in which the former was to estimate the effect of instrumental variables on product quality certification. The results were also carried over to the degree of green production transition equation to estimate the impact of product quality certification on green production transition.

### 3.4. Multi-Valued Treatment Effect Model

To further analyze the differential impact of participation in different product quality certifications on kiwifruit growers’ green production transition, we employed the multi-valued treatment effect model in our analysis [46] in that product quality certifications contain four main modes: no certification, pollution-free certification, green certification, and organic certification. The equation of the multi-valued treatment effect model is as follows:

(4) $$y_{i}=\sum_{m=0}^{M}D_{im}(T_{i})y_{im}$$

where, $D_{im}(T_{i})$ is the indicator variable for the selection of the m-th treatment state by the i-th kiwifruit growers. When $T_{i}=m$, $D_{im}(T_{i})=1$; else, $D_{im}(T_{i})=0$. When $T_{i}=m$, the potential outcome variable corresponding to the i-th kiwifruit farmer is $y_{m}$. Accordingly, the conditional expectation of the transition probability of green production of kiwifruit growers can be obtained and expressed as:

(5) $$E[y_{im}|Z_{i}]=E[y_{im}|T_{i}=m,Z_{i}]=\beta_{0m}+Z_{i}\beta_{1m}$$

where, $\beta_{m}=[\beta_{0m}\;\beta_{1m}]$ is the parameter to be estimated. It is worth noting that the multi-valued treatment effect model requires generalized propensity value (GPS) regression adjustment to calculate the conditional expectations of the equations of the outcome variables corresponding to the different treatment state $T_{i}$. The equation for the generalized propensity value $r_{i}$ is given by:

(6) $$r_{i}=(m,Z)=\mathrm{Pr}[T_{i}=m|Z_{i}]=E[D_{im}(T_{i})|Z_{i}]$$

In summary, we employed the inverse probability weighted regression adjustment method to estimate the average treatment effect (ATE) [47]. The equation of the average treatment effect for the full sample and subsamples is as follows:

(7) $$ATE_{mk}=({\hat{\beta }}_{0m}-{\hat{\beta }}_{0k})+\frac{1}{N}\sum_{i=1}^{N}Z_{i}({\hat{\beta }}_{1m}-{\hat{\beta }}_{1k})$$

(8) $$ATET_{mk}=({\hat{\beta }}_{0m}-{\hat{\beta }}_{0k})+\frac{1}{N_{m}}\sum_{i:D_{t}(T_{i}=m)=1}^{N}Z_{i}({\hat{\beta }}_{1m}-{\hat{\beta }}_{1k})$$

### 3.5. Data

#### 3.5.1. The Sample

The research team’s field survey data on farmers in the main kiwifruit-producing areas in Sichuan and Shaanxi provinces from September to October 2018 were used. These two provinces were taken as subject provinces because they are the top two provinces in China in terms of kiwifruit planting area, and their kiwifruit planting area accounts for about 60% of China’s total kiwifruit planting area. Kiwifruits in Shaanxi Province are mainly geographically distributed in Zhouzhi County, Meixian County, and Wugong County, while involving relatively more areas in Sichuan Province. In this case, we selected Dujiangyan City, Cangxi County, and Pujiang County as our object regions. The survey was based on stratified, multi-stage, and proportional to size probability sampling (PPS) methods [48], during which a combination of stratified and random sampling was used, and the specific sampling process was as follows. First, we selected three to five townships according to the size of the kiwifruit industry in the county (city); second, we randomly selected 3–5 villages in each township, and 8–12 kiwifruit growers in each village. Finally, we selected one member of each sampled household who was familiar with their household situation in our survey. The survey process followed the principle of combining stratified sampling and random sampling, covering 110 villages in total. After excluding invalid questionnaires such as unanswered key variables and subjects who did not meet the requirements, we collected 1036 valid questionnaires.

#### 3.5.2. Variables Measuring Green Agricultural Production Transition

We constructed a latent class stochastic frontier model that was based on the C-D production function describing the input-output relationship of kiwifruit growers with the equation.

(9) $$Y_{i}=AK_{i}^{\alpha }L_{i}^{\beta }e^{\mu }$$

where, $Y_{i}$ is the annual income per mu of kiwifruit for i-th kiwifruit grower, $K_{i}$ is the capital input per mu of kiwifruit for i-th kiwifruit grower, $L_{i}$ is the labor input per mu of kiwifruit for i-th kiwifruit grower, A is the comprehensive technology level, and μ is the random error term. Logarithmizing Equation (9), we obtained the following Equation (10):

(10) $$\ln Y_{i}=\ln A+\alpha \ln K_{i}+\beta \ln L_{i}+\mu$$

The relevant covariates were selected in the finite mixture model based on the requirements of the Key Points of Planting Industry in 2020 that was issued by the Ministry of Agricultural and Rural Affairs of China and combined with kiwifruit planting characteristics. We selected five indicators of organic fertilizer application rate, biological pesticide use rate, input in water-saving irrigation equipment, input in physical control technology, and packaging recycling rate as important ways to judge kiwifruit growers’ production methods. The five indicators are only a few performance characteristics of farmers’ green production methods and do not fully represent those of kiwifruit growers. Using the relationship between these closely related covariates and output, the probability of kiwifruit growers adopting green production practices can, however, be calculated indirectly, thus obtaining the proxy variables for green production transition. The meanings of the relevant variables are shown in Table 1.

#### 3.5.3. Core Explanatory Variables

The main types of agricultural product quality certification in China are pollution-free agricultural product certification, green food certification, and organic agricultural product certification. In this study, participation in agricultural quality certification was characterized by the following question: “Does the household participate in at least one of the certifications of pollution-free agricultural products, green food, organic agricultural products, or geographical indications for agricultural products?” If the answer is “yes”, we confirm that the household was involved in agricultural product quality certification, otherwise it would be not. Also, we measured the types of agricultural quality certification. If a kiwifruit grower was not involved in agricultural quality certification, the value would be 0. If a kiwifruit grower has been certified as pollution-free, the value would be 1. If a kiwifruit grower has passed the green food certification, the value would be 2. If a kiwifruit grower has obtained the organic agricultural product certification, the value would be 3. In order to examine the net processing effect of product quality certification on the transition of production methods and to avoid multiple product quality certification samples from interfering with the regression results, we excluded farmers who have passed multiple product quality certifications simultaneously. Finally, we got valid questionnaires from 974 kiwifruit growers.

#### 3.5.4. Control Variables

In line with the existing literature and our model selection procedures, we selected variables that affect the transition of green production of farmers, such as the characteristics of individuals, of family, of social networks, and of government support, as control variables [2,49]. For individual characteristics, the age and the education level of the household head were selected. For household characteristics, the scale of operation, planting specialization, and the number of agricultural training were selected; social network characteristics included the presence of village cadres in the household and the expenditure on human interaction; for government support, the green publicity efforts of the local government were selected; and for regional variables, the distance from the township and province were selected.

## 4. Results and Discussion

### 4.1. Results of the Measurement of Green Agricultural Production Transition

#### 4.1.1. Determining the Categories of Production Methods of Kiwifruit Growers

Model estimation started with two categories and was repeated for an ever-increasing number of categories. To ensure that the size of each category remains meaningful for interpretation given the sample size, we suspended the procedure after three classes. We chose the appropriate number of categories based on how different models scored regarding model fit and various information criteria. According to the results of the finite mixture model in Table 2, the Bayesian information criterion (BIC) value was 177.004 when the number of categories was 2, which was lower than the BIC values when that was 1 and that was 3, respectively. Therefore, it is determined that it was appropriate to classify kiwifruit growers’ production methods into two categories.

#### 4.1.2. Probability Analysis of Samples Falling into Potential Categories

We collapsed the probability of the sample kiwifruit growers falling into the two categories to decompose the analysis to the probability of falling into Class A (the probabilities of falling into Class A and Class B were the same). The study showed that of the 974 samples, 206 kiwifruit growers (21.15% of the total sample) had a posterior probability that was greater than 0.5 with a mean probability of 0.773, and 768 kiwifruit growers had posterior probabilities that were less than or equal to 0.5 with a mean probability of 0.253. To compare the differences in covariates between the two groups of growers in Class A and Class B, we identified them using a sample mean *t*-test. The results in Table 3 show that the means of each covariate in Class A are significantly higher than those in Class B, indicating that the adoption of green production practices was more pronounced among farmers in Class A. The posterior probability of falling into Class A that was calculated by the finite mixture model was thus employed to measure the degree of green production transition among kiwifruit growers.

### 4.2. Estimation Results of Whether to Participate in Agricultural Product Quality Certification on Green Production Transition

In verifying the impact of product quality certification on kiwifruit growers’ green production transition, product quality certification may result from self-selection by kiwifruit growers. The variable product quality certification may not satisfy random sampling, and the direct regression may endogenize the estimation results due to non-random sampling. In order to ensure unbiased model estimation results, the CMP method was used to solve the endogeneity problem. We employed the leave-one-out method to select the average level of certification as the instrumental variable. Then, it was measured by the proportion of the number of households in the village with product quality certification, other than their households to the number of farm households that there were. The following two conditions are indispensable for the instrumental variables that were selected for the study. On the one hand, they are correlated with the product quality certification as the explanatory variable (correlation assumption), but on the other hand, they are not correlated with the error term of the model (exclusivity assumption). As a typical acquaintance society, Chinese villages have a high degree of trust and identity within the village group due to the close kinship and local bonding [50]. There is a strong correlation between the product quality certification of a farmer’s household and the product quality certification of others in the same village, but the average status of product quality certification of other individuals in the village does not directly affect the household’s green production transition.

Column (1) of Table 4 shows the regression results of product quality certification on the degree of green production transition of kiwifruit growers that were estimated by the CMP model [51]. It can be inferred from the results that in the two-stage instrumental variables estimation, the LR test value of 134.58 that was estimated by the one-stage model rejected the null hypothesis and ruled out the possibility of weak instrumental variables. Moreover, the average level of certification was highly correlated with the endogenous variable of product quality certification, so the instrumental variable selection is feasible. In addition, the average level of certification passed the test at 1% significance level in the selection equation, and so was the product quality certification at the same significance level in the second-stage outcome equation. This suggests that product quality certification can still significantly contribute to the green production transition of kiwifruit growers when the endogeneity problem is addressed. To further verify the robustness of the regression results, kiwifruit growers with a degree of green production transition that was greater than 0.5 were assigned 1 for achieving green production transition and 0 for those that had not. We utilized the Endogenous Switching Probit (ESP) model for further robustness testing by treating the degree of green production transition as a dichotomous choice variable as to whether or not they transitioned to green production as described above. As shown in Table 4, the estimation results explained that the Wald test value is 101.77, which rejects the original hypothesis passing the test at the 1% significance level. Therefore, the ESP model is appropriate to deal with the endogeneity due to selection bias. In addition, the sign of variables in the selection and outcome equations remained broadly consistent with the estimation results of the CMP model, making it clear that the estimation results of the baseline regression are more robust.

### 4.3. Estimation Results of Participation in Different Types of Agricultural Product Quality Certification on Green Production Transition

#### 4.3.1. Overlap Assumption Testing

**Figure 2:**
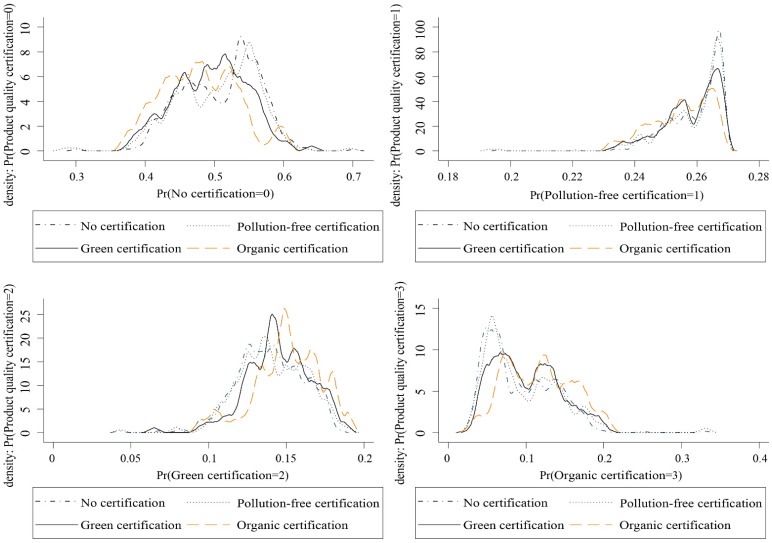
Conditional probability distribution of agricultural product quality certification.

We employed a multi-valued treatment effect to test for the differences in the degree of green production transition among kiwifruit growers in different certification types. Before that, however, we need to conduct overlap assumption testing, and the overlapping assumption testing or conditional independence assumption served as a prerequisite for the multi-value treatment effect model analysis [52]. Our findings described that the conditional probabilities of pollution-free certification, green certification, and organic certification all ranged from 0 to 1 with a significant overlap interval, which illustrates that the multi-valued treatment effect analysis can be performed accordingly. The conditional probability distribution is shown in Figure 2.

#### 4.3.2. Result Analysis of Different Certification Types on the Green Production Transition

The regression results of the factors influencing the green production transition of the total sample of kiwifruit growers are shown in column (1) of Table 5. The education level of household head, planting specialization, number of agricultural trainings, and expenditure on human interaction all significantly enhancing the green production transition of kiwifruit growers, and the scale of operation significantly inhibited the degree of green production transition of kiwifruit growers. Regarding the different types of certifications and for kiwifruit growers without product quality certification, the higher the education level of household head and the degree of planting specialization, the higher the probability of green production transition by kiwifruit growers. For kiwifruit growers without pollution-free certification, the smaller the scale of operation, the higher the degree of planting specialization, and the greater the expenditure on human interaction, thus bringing about a higher probability of green production transition of kiwifruit growers. For green-certified kiwifruit growers, a significant inhibitory effect of scale of operation on the degree of green production transition of kiwifruit growers was thus seen. In contrast, planting specialization, village cadres in the household, and personal expenses all contributed to the green production transition of kiwifruit growers. For the kiwifruit growers of organic certification, the scale of operation had a significant inhibitory effect on the degree of green production transition of kiwifruit growers. The degree of planting specialization promoted the green production transition of kiwifruit growers. In summary, the factors affecting the green production transition of kiwifruit growers were significantly different in respect of different certification types.

#### 4.3.3. Average Treatment Effect of Quality Certification Types on Green Production Transition

The inverse probability weighted regression adjustment (IPWRA) method was utilized to calculate the average treatment effect (ATE) of different certification types on kiwifruit growers’ green production transition. The results accounted for that the probability of green production transition was significantly higher for kiwifruit growers with pollution-free, green, and organic certification than for those without certifications (ATE was 0.0629, 0.1870, 0.4533, respectively), and all passed the test at 1% significance level. This indicates that product quality certification can significantly contribute to green production transition of kiwifruit growers. In addition, in terms of the average treatment effect of different certification types, the promotion effect of organic certification on growers’ green production transition was greater than that of both the green and pollution-free certification, which is mainly arranged as organic > green > pollution-free certifications. What we can infer from the results is a significant difference in the effect of different quality certification methods on green production transition for growers. It could be that the standards of organic certification are higher than those of green and pollution-free certifications for the time being, and organic certification stipulates that the use of pesticides and chemical fertilizers is not allowed, but the green certification does. The regulations on the dosage and residue levels for green certification are, however, stricter than those for pollution-free certification in general, so organic certification is of a more substantial binding effect on growers’ green production behavior, followed by green certification and pollution-free certification. To further verify the robustness of the results, the dependent variable was treated as “has the kiwifruit farmer achieved a green production transition” following the treatment above. We obtained similar results as well.

### 4.4. Mechanisms of Impact of Product Quality Certification on Green Production Transformation of Kiwifruit Growers

The baseline regression results displayed that product quality certification can significantly contribute to the green production transition of kiwifruit growers, but the channels through which product quality certification affects the kiwifruit growers’ green production transition are more worthy of attention. Combined with the previous theoretical analysis, we intended to examine three mechanisms, namely quality monitoring, product premium, and market access threshold, to systematically investigate the transmission channels through which product quality certification influences the green production transition of kiwifruit growers. Among them, the quality monitoring variable is “the degree of strictness of kiwifruit product testing by regulatory testing agencies: not strict = 1; not too strict = 2; average = 3; relatively strict = 4; very strict = 5”. The higher the value, the stricter the quality monitoring. The product premium variable was mainly characterized by “the average unit price of your kiwifruit marketed in 2018 (unit: yuan/catty)”. The market access threshold was mainly expressed as “how easy is it for your kiwifruit to enter the modern sales market (supermarkets or micro-business such as Taobao stores): very difficult = 1; relatively difficult = 2; average = 3; relatively easy = 4; very easy = 5”, with larger values indicating a smaller market access threshold. In this regard, this paper applied a mediating effect model to test the mechanism, considering the characteristics of the independent, mediating, and dependent variables as categorical variables. Then, we used the mediating effect model to test the channels. Considering the characteristics of the independent, mediating, and dependent variables as categorical variables, however, we adopted the Iacobucci test to construct the $Z_{mediation}$ statistic for the mediating effect test [53]. The mediating effect is considered significant if $Z_{mediation}>1.96$ at the significance level of 0.05.

According to the results of the channel test shown in Table 6, column (1) explains that the coefficient of influence of product quality certification on quality monitoring is positive, passing the test at 1% significance level; column (2) of Table 6 manifests that the coefficient of influence of quality monitoring on green production transition is positive, passing the test at 1% significance level. Besides, the regression coefficient of product quality certification becomes gradually smaller, but the value of $Z_{mediation}$ is more significant than 1.96. It can be seen from the above regression result that quality monitoring plays a part in mediating the effect of product quality certification on green production transition of growers, which verifies the existence of the quality monitoring influence mechanism. The regression results in columns (3) and (4) of Table 6 accounted for that the coefficients of the effect of product quality certification on product premium, and of product premium on green production transition, are both positive and pass the test at 1% significance levels, respectively. The value of $Z_{mediation}$ is 6.142 greater than 1.96, demonstrating that the mediating effect of product premium also comes into play. The above results verify the existence of the product premium mechanism. Further, the regression results in columns (5) and (6) of Table 6 indicate that the effect of product quality certification on market access threshold, and of market access threshold on green production transition, are both positive and pass the test at 1% significance levels respectively. The value of $Z_{mediation}$ is 6.676 greater than 1.96, manifesting that the market access threshold plays a part in the mediating effect. The above results verify the existence of the market access threshold mechanism. In conclusion, product quality certification significantly influences the green production transition through quality monitoring, product premium, and market access threshold, and Hypothesis 2 is thus verified.

## 5. Conclusions

As an essential means to realize the development of green agriculture and overcome environmental problems in agricultural practices, the transition of agricultural green production has been increasingly discussed, especially for China with extensive agricultural production. In this research, we build on input-output data from kiwifruit growers in Shaanxi and Sichuan provinces to measure the degree of green production transition of kiwifruit growers with the help of a finite mixture model. Then, the effect of product quality certification on green production transition of kiwifruit growers was examined based on an econometric analysis model. Using a multi-valued treatment effects model, we also examined the differences in green production transition among kiwifruit growers under various certification types. Finally, the channels of influence of product quality certification on kiwifruit growers’ green production transition are analyzed by mechanism testing.

Our findings are as follows. First, the degree of green production transition among kiwifruit growers is generally not that high, on an average of only 36.3%, so there is a lot of room for improvement in this respect. Second, product quality certification significantly propels the green production transition of kiwifruit growers; this finding remains robust after considering the endogeneity of selection bias. Product quality certification can advance the green production transition of kiwifruit growers through three mechanisms: quality monitoring, product premium, and market access threshold. Third, there is significant heterogeneity in different product quality certifications in boosting the green production transition of kiwifruit growers, with organic certification playing the most significant role, followed by green certification then pollution-free certification.

The main research limitations of this paper are the following three aspects. Firstly, we used five variables of organic fertilizer application rate, biological pesticide use rate, input in water-saving irrigation equipment, input in physical control technology, and packaging recycling rate as proxy variables for farmers’ green production transition, which may not be comprehensive, and subsequent studies need to include more parameter variables to make the model assumptions more realistic. Secondly, the data that we collected through the questionnaire survey are static cross-sectional data, and it is difficult to reveal the dynamic influence mechanism of these variables using these data. Therefore, in future research, the effect of product quality certification on farmers’ green production transition can be systematically investigated by collecting time series data or survey data in a larger area. Thirdly, although the sample was selected from farmers in the typical kiwifruit production areas in China, the sampling range is still relatively small. Therefore, in the future, we will expand the scope of the questionnaire and the sample size to cover different types of farmers and different agricultural producers at different levels, and on this basis, we will conduct more relevant research.

## Figures and Tables

**Table 1:** Descriptive statistics for measuring the main variables of green agricultural production transition.

	Variables	Description
Input-output variables	Output of kiwifruits	Annual income of kiwifruits per acre (yuan/mu ^a^)
	Capital input	Total physical capital and mechanical capital input per acre of kiwifruits (yuan/mu)
	Labor input	Total average labor input per mu for each production link of kiwifruits (yuan/mu)
Covariates of the finite mixture model	Organic fertilizer application rate	Proportion of total fertilizer application costs for kiwifruit growers using organic fertilizers (%)
	Biopesticide usage rate	Kiwifruit growers’ cost of using biopesticides as a percentage of total pesticide use (%)
	Water-saving irrigation equipment utilization rate	Proportion of kiwifruit growers using water-saving irrigation equipment such as drip irrigation and sprinkler irrigation to total scale of operation (%)
	Input in physical control technology	Inputs per mu of physical control techniques used by kiwifruit growers in the production process of kiwifruits, including physical control techniques such as insect trap lights and insecticidal boards (yuan/mu)
	Packaging recycling rate	Recycling rate of pesticide and fertilizer packaging used in kiwifruit production by kiwifruit growers (%)

^a^ One yuan was equal to 0.15 US dollars in April 2022; one mu is equal to 0.067 hectares.

**Table 2:** The results of determining the potential categories to which the production methods belong.

Number of Categories	Log-Likelihood Value	Number of Parameters	BIC
1	−78.930	7	260.031
2	−36.892	15	177.004
3	−25.630	23	209.532

**Table 3:** Comparison of the main indicators of green production transition of potential categories.

Variables	Class A		Class B		Difference	T-Value
	Sample	Mean	Sample	Mean		
Percentage of organic fertilizer	206	54.204	768	50.141	4.064 **	2.154
Biopesticide usage rate	206	71.408	768	66.953	4.455 **	2.114
Input in water-saving irrigation equipment	206	42.379	768	36.484	5.894 **	2.062
Input in physical control technology	206	116.505	768	84.375	32.130 ***	2.608
Packaging recycling rate	206	48.141	768	44.850	3.291 **	2.425

Note: ***, ** denote values significant at the 1% and 5% levels, respectively.

**Table 4:** Estimation results of endogeneity tests.

Variables	(1) CMP Model		(2) ESP Model		
	Product Quality Certification	Green Production Transition	Product Quality Certification	Is Green Production Transition Implemented?	
				Yes	No
Product quality certification		0.395 *** (0.030)			
Average degree of certification	7.193 *** (0.559)		7.243 *** (0.773)		
Constant	−4.160 *** (0.530)	0.061 (0.083)	−4.240 *** (0.613)	−0.027 (0.605)	−2.758 *** (0.882)
Control variables	Controlled	Controlled	Controlled	Controlled	Controlled
atanhrho_12	−0.765 *** (0.095)				
Residual correlation coefficient				*ρ*_1_ = −0.687	*ρ*_0_ = −0.084
Wald test values			101.77 ***		
LR test value	134.58				
Samples	974		974		

Note: *** indicates significant at the 1% level; robust standard errors are in parentheses.

**Table 5:** Estimation results of the green production transition for kiwifruit grower’s certification types.

Variable	Full Sample	No Certification	Pollution-Free Certification	Green Certification	Organic Certification
Age	−0.001	−0.000	−0.002	−0.001	0.004
	(0.001)	(0.001)	(0.002)	(0.003)	(0.003)
Education	0.007 **	0.006 **	0.000	−0.006	0.012
	(0.003)	(0.003)	(0.005)	(0.007)	(0.009)
Business scale	−0.015 ***	−0.002	−0.014 ***	−0.027 ***	−0.016 **
	(0.003)	(0.004)	(0.004)	(0.006)	(0.008)
Planting specialization	0.006 ***	0.002 **	0.006 ***	0.004 **	0.009 ***
	(0.001)	(0.001)	(0.001)	(0.002)	(0.002)
Number of training	0.026 **	0.007	0.027	0.021	0.004
	(0.011)	(0.011)	(0.020)	(0.030)	(0.034)
Village cadres	0.012	−0.030	−0.006	0.271 ***	0.044
	(0.027)	(0.024)	(0.043)	(0.082)	(0.069)
Personal expenses	0.119 **	−0.030	0.145*	0.323 **	0.242
	(0.056)	(0.060)	(0.085)	(0.144)	(0.156)
Distance to township	−0.006	−0.009	−0.029	−0.008	0.039
	(0.014)	(0.013)	(0.027)	(0.044)	(0.049)
Government green promotion	0.010	0.010	0.015	0.014	0.006
	(0.006)	(0.007)	(0.012)	(0.017)	(0.016)
Province	−0.031	0.052 **	−0.044	−0.018	−0.137 ***
	(0.022)	(0.023)	(0.041)	(0.050)	(0.050)
Constant	0.222 ***	0.152 *	0.298 *	0.440 *	0.096
	(0.077)	(0.079)	(0.154)	(0.238)	(0.213)
Sample	974	490	252	138	94

Note: *, ** and *** denote significant at the 10%, 5% and 1% levels, respectively; robust standard errors are in brackets.

**Table 6:** Regression results for the channel test.

	(1)	(2)	(3)	(4)	(5)	(6)
	Quality Monitoring	Green Production Transition	Product Premium	Green Production Transition	Market Access Threshold	Green Production Transition
Product quality certification	0.634 ***	0.139 ***	0.923 ***	0.133 ***	0.664 ***	0.146 ***
	(0.072)	(0.015)	(0.114)	(0.014)	(0.072)	(0.015)
Quality monitoring		0.031 ***				
		(0.007)				
Product premium				0.025 ***		
				(0.004)		
Market access threshold						0.016 **
						(0.007)
Control variables	Controlled	Controlled	Controlled	Controlled	Controlled	Controlled
Constant	-	0.049	4.486 ***	0.045	-	0.107
		(0.078)	(0.603)	(0.076)		(0.076)
Iacobucci Test	6.364		6.142		6.676	
R^2^ (Pseudo R^2^)	0.042	0.207	0.329	0.226	0.046	0.198
Sample	974	974	974	974	974	974

Note: **, *** denote significant at the 5%, and 1% levels, respectively; robust standard errors are in brackets.

## Data Availability

The data will be provided upon request to the corresponding author. The datasets used and/or analyzed during the current study are available from the corresponding author on reasonable request.
